# Jointly Optimization of Delay and Energy Consumption for Multi-Device FDMA in WPT-MEC System

**DOI:** 10.3390/s24186123

**Published:** 2024-09-22

**Authors:** Danxia Qiao, Lu Sun, Dianju Li, Huajie Xiong, Rina Liang, Zhenyuan Han, Liangtian Wan

**Affiliations:** 1 Department of Communication Engineering, Institute of Information Science Technology, Dalian Maritime University, Dalian 116026, China; qiaodanxia63@dlmu.edu.cn (D.Q.); rinaliang@dlmu.edu.cn (R.L.); han1120221370@dlmu.edu.cn (Z.H.); 2 State Key Laboratory of Satellite Navigation System and Equipment Technology, The 54th Research Institute of China Electronics Technology Group Corporation, Shijiazhuang 050081, China; huajiexiong206@163.com; 3 The 723 Institute of China State Shipbuilding Corporation Limited, Yangzhou 101400, China; galaxy0202li@163.com; 4 Key Laboratory for Ubiquitous Network and Service Software of Liaoning Province, School of Software, Dalian University of Technology, Dalian 116620, China; wanliangtian@dlut.edu.cn

**Keywords:** WPT-MEC, resource scheduling, Q-learning, immune algorithms

## Abstract

With the rapid development of mobile edge computing (MEC) and wireless power transfer (WPT) technologies, the MEC-WPT system makes it possible to provide high-quality data processing services for end users. However, in a real-world WPT-MEC system, the channel gain decreases with the transmission distance, leading to “double near and far effect” in the joint transmission of wireless energy and data, which affects the quality of the data processing service for end users. Consequently, it is essential to design a reasonable system model to overcome the “double near and far effect” and make reasonable scheduling of multi-dimensional resources such as energy, communication and computing to guarantee high-quality data processing services. First, this paper designs a relay collaboration WPT-MEC resource scheduling model to improve wireless energy utilization efficiency. The optimization goal is to minimize the normalization of the total communication delay and total energy consumption while meeting multiple resource constraints. Second, this paper imports a BK-means algorithm to complete the end terminals cluster to guarantee effective energy reception and adapts the whale optimization algorithm with adaptive mechanism (AWOA) for mobile vehicle path-planning to reduce energy waste. Third, this paper proposes an immune differential enhanced deep deterministic policy gradient (IDDPG) algorithm to realize efficient resource scheduling of multiple resources and minimize the optimization goal. Finally, simulation experiments are carried out on different data, and the simulation results prove the validity of the designed scheduling model and proposed IDDPG.

## 1. Introduction

With the rise of the Internet of Everything, network edge devices continue to generate rich data resources. However, the rapid growth of these data volumes far exceeds the processing capacity of traditional cloud computing centers, presenting challenges in bandwidth and latency. In the centralized big data processing mode of the cloud computing era, data are destined for a long-distance trip to the central server, which not only increases the burden of the network but also struggles to satisfy the need for immediacy [1]. To address these issues, it has become an effective solution to migrate the tasks that originally need to be transmitted to cloud computing to the edge cloud near the device terminal for processing. Edge computing streamlines data processing progress by establishing computational nodes at the network’s edge, thereby reducing the delay of data transmission and network congestion [2]. With a distributed computing setup, the system achieves enhanced data transmission performance and assures on-time data processing so that the application can respond quickly and provide users with a smoother experience.

Given the limited battery of edge devices, the edge device needs to charge its battery after a period of power-on. Therefore, how to ensure economical and sustainable power for numerous Internet of Things (IoT) devices is a major challenge. We can use a wired way to charge it, as exemplified by mobile phones, which can be recharged using a charger once depleted. However, this method restricts the user’s mobility and is impractical in many scenarios, such as in wireless sensor networks where charging interfaces are often inaccessible [3]. To overcome these limitations, the fusion of wireless power transfer (WPT) technology with mobile edge computing (MEC) presents an innovative approach, culminating in the development of the WPT-MEC system [4]. The WPT-MEC system supplies energy to edge devices wirelessly and uses MEC’s computing capabilities to implement data computation at the network’s edge. This approach not only mitigates data transmission delays but also enhances the system’s response speed and overall efficiency.

However, we observe a “double near and far effect” in the multi-node WPT-MEC system [5] if the energy station and the edge cloud server are configured together. Under the influence of this effect, terminal devices close to the energy station enjoy better channel conditions. However, this results in terminal devices farther away from the energy station collecting less energy and consuming more to communicate with the edge cloud, which is obviously unfair to distant devices. Such unfairness can compromise the overall system performance, as distant devices may fail to complete their tasks due to energy scarcity. Additionally, it may increase the system’s complexity and costs, necessitating frequent energy replications.

To address this challenge, the WPT-MEC system must develop an efficient resource allocation method. This method should consider the distribution of computing and communication resources while balancing the energy supply [6]. It involves intelligent scheduling of energy emitters, the dynamic allocation of edge computing tasks, and the optimization of device energy harvesting and consumption behaviors. For example, the energy harvesting efficiency of remote devices can be enhanced by adjusting the power or frequency of the energy transmitters. Additionally, optimizing the task allocation algorithm can ensure that computing tasks are preferentially assigned to devices with sufficient energy supply.

In summary, the search for an efficient resource allocation method for the WPT-MEC system has garnered extensive attention from academic communities worldwide. System performance indicators are critical, such as total task processing delay and equipment energy efficiency. Reinforcement learning algorithms, known for their efficiency and robustness, have attracted significant interest in solving complex optimization problems, especially in high uncertainty and multi-parameter search spaces. These algorithms hold the potential for dynamic resource allocation in the WPT-MEC system. Despite the extensive focus on task processing delay and equipment energy efficiency within the WPT-MEC system, the integration of reinforcement learning algorithms with cooperative resource allocation methods remains understudied [7]. Complexity abounds in the realm of resource allocation for the WPT-MEC system, necessitating consideration of the stochastic and time-varying nature of energy harvesting, along with the diversity and real-time demands of computing tasks. Moreover, the design of reinforcement learning algorithms should consider convergence speed, computational complexity, and adaptability to environmental changes [8]. Therefore, designing a reinforcement learning algorithm that effectively minimizes task-processing delay, enhances equipment energy efficiency, and demonstrates strong adaptability is a pressing area of research within the WPT-MEC system.

This article introduces a model for a dynamic wireless-powered edge computing framework. In this model, a mobile vehicle equipped with an edge computing server and a directional wireless power supply device offers directional wireless charging and computing, offloading services to terminal devices. Note that the charging station in this model has a stable power supply, eliminating concerns regarding the continuous power supply of the mobile vehicle. Our objective is to ensure the system operates efficiently and economically by minimizing the weighted combination of task execution delays and power consumption for end-devices. Our contributions are as follows:

(1) To address the dual near-far effect in the system, a scheme for collaborative offloading among terminal devices is proposed. Frequency-division multiple access (FDMA) technology facilitates the simultaneous offloading for various terminal devices.

(2) Considering the random distribution of terminal devices in the scene, we first employ the K-means algorithm to organize devices into clusters by their physical locations. In the device clustering preprocessing phase, we improve the K-means algorithm by incorporating a binary search-based approach for *K* selection, resulting in a modified version termed the BK-means algorithm. Following the completion of clustering, we utilize a hybrid Whale Optimization Algorithm (WOA) with an adaptive strategy to rationally plan the overall driving path of the vehicle.

(3) We establish a mathematical model aimed at minimizing the weighted combination of task execution delays and power consumption for terminal devices. By leveraging the strengths of the differential immune process in conjunction with the Deep Deterministic Policy Gradient (DDPG) algorithm, we introduce an Immune differential-enhanced Deep Deterministic Policy Gradient (IDDPG) algorithm. This algorithm is designed to address the optimization objective and achieve optimal resource scheduling within a dynamic wireless-powered edge computing system under multi-dimensional constraints.

(4) We have devised simulation experiments to assess the efficacy of the IDDPG algorithm. The IDDPG algorithm demonstrates faster convergence and significantly lower system overhead compared to the traditional DDPG algorithm. Moreover, it identifies superior resource scheduling strategies within the dynamic wireless-powered edge computing system.

The paper is organized as follows: Section 2 reviews the related work in this field. Section 3 presents the multi-device relay cooperation WPT-MEC system model. Section 4 describes the enhanced IDDPG algorithm. Section 5 details the simulation experiments. Section 6 marks the conclusion of the paper and proposes future research endeavors.

## 2. Related Work

WPT greatly improves the convenience and flexibility of energy supply by transmitting electrical energy wirelessly. MEC offloads computing tasks to closer edge devices, which is essential for reducing transmission delay and improving computing efficiency. In the field of resource optimization, evolutionary algorithms have proved their effectiveness in solving complex problems. However, as the system environment becomes more dynamic and uncertain, traditional evolutionary algorithms may have limitations in real-time adaptability. In this context, reinforcement learning (RL), especially deep reinforcement learning (DRL), has begun to attract attention due to its potential to overcome these limitations.

### 2.1. WPT

Energy harvesting can be approached through a multitude of methods. Wei et al. introduced several prevalent energy harvesting models that are commonly utilized, conducting an in-depth analysis of the resource allocation challenges in simultaneous wireless information and power transfer (SWIP) systems [9]. Wang et al. integrated non-orthogonal multiple access (NOMA ) technology into Multiple-Input Multiple-Output (MIMO) networks, thereby enhancing spectral and energy efficiency [10]. At the same time, for the complexity of the resource allocation problem, the Alternating Direction method of Distributed Multipliers (ADMM) algorithm was proposed. Feng et al. delved into the complex issues of energy consumption in WPT-assisted federated learning systems [11]. Xu et al. studied the energy consumption of WPT-backscatter communication networks and took into account the dynamic reflection coefficient [12]. Bai et al. analyzed and studied the energy consumption of WP-MEC systems integrated with an intelligent reflecting surface (IRS) [13]. IRS can solve the problem of channel attenuation for wireless devices. Zhu et al. considered the same system as the former, except that Zhu et al. focused on the throughput problem [14]. Feng et al. have conducted an in-depth investigation into the energy harvesting problem specific to WPT networks for single unmanned aerial vehicles [15]. In contrast to the aforementioned study on single-UAV scenarios, Luo et al. studied multi-UAV WPT networks and considered the probabilistic line-of-sight channel model [16].

### 2.2. MEC

To tackle the issue of task and resource allocation in MEC networks, Wang et al. have introduced a multi-stack reinforcement learning approach. Logging the historical user status and resource allocation has been implemented to avoid the repetitive learning of identical schemes, which results in improved convergence rates and learning productivity [17]. Huang et al. executed an analysis on the intricate issues of dynamic admission control and resource distribution within MEC-enhanced small-cell networks (SCNs) [18]. They developed the Admission Control and Computation Resource Allocation (ACCRA) algorithm to determine the most efficient resolutions for the sub-problems on a distributed basis [19] and studied multi-hop techniques in SCNs, considering the implementation of NOMA in MEC and the allocation of heterogeneous computing resources. A dynamically optimized model was suggested to optimize the overall energy efficiency of uplink and downlink transmissions. Li et al. integrated UAVs as cloud nodes to optimize their energy efficiency by concurrently refining the UAVs’ flight paths, user transmission power, and computation load distribution [20]. Considering the energy consumption caused by task offloading in the MEC system, Jiang et al. proposed an online framework for joint offloading and resource allocation under energy constraints, employing Lyapunov optimization to convert long-term constraints into immediate problems [21]. In addition, Bahreini proposed G-ERAP and APX-ERAP as two allocation and pricing mechanisms, considering the dynamic supply of computing resources. The economic efficiency of the mechanism is ensured by collecting user requests and sorting them by bid density [22]. Considering the mobility of ground users, Ref. [23] proposed MEC based on D2D cooperation and constructed a task scheduling framework based on user mobility to minimize the task offloading delay. Liang et al. designed practical algorithms for optimal joint migration/switching strategies [24]. To mitigate service disruptions from user movement, a solution method based on effective relaxation and rounding is developed by jointly managing computing resources and radio resources.

### 2.3. DRL

Recent advancements in MEC, unmanned aerial vehicle (UAV)-assisted networks, and resource allocation have garnered significant research attention, driven by their potential to amplify the performance of modern wireless networks.

In the realm of MEC, the work by [25] presented a DRL framework that addressed the challenge of combined task offloading and resource allocation under dynamic conditions. The proposed actor–critic learning structure effectively optimized both the determination of task offloading and the distribution of CPU resources, achieving near-optimal performance while reducing computational complexity. Complementing this, Ref. [26] tackled resource distribution in IoT edge networks through an improved Deep Q-Network (DQN) algorithm. This approach leveraged multiple replay memories and decoupled job scheduling from resource adjustment to enhance convergence and reduce action space complexity.

The integration of UAVs into MEC systems has also been extensively studied. The paper by [27] formulated a mixed-integer nonlinear programming (MINLP) problem for optimizing UAV positions and UAV–UE association in a cooperative network. To address non-convexities and the challenge of lacking channel state information (CSI), a novel algorithm combining deep Q-learning with a difference of convex functions approach was proposed. Similarly, Ref. [28] explored UAV-assisted MEC networks where tasks were offloaded to UAVs collaborating with access points, demonstrating significant gains in system performance through collaborative processing.

Additionally, the deployment of UAVs in IoT networks has been investigated. In [29], a resource distribution strategy for IoT edge networks was designed to minimize the weighted aggregate of job completion times and requested resources. The improved DQN algorithm used here outperformed traditional methods by enhancing convergence and efficiency. Meanwhile, Ref. [30] examined UAV-aided networks for NOMA uplink transmissions, optimizing UAV height and channel assignments to boost system capacity.

In the context of high-mobility networks, Ref. [31] proposed a DRL-based smart optimization algorithm for 5G networks to dynamically allocate resources and improve throughput and packet loss rates. This approach utilized deep neural networks to adaptively adjust TDD ratios in response to dynamic traffic conditions. On the other hand, Ref. [32] addressed security and privacy issues in edge-centric IoT environments by integrating blockchain technology. The proposed framework employs smart contracts with an asynchronous advantage actor–critic (A3C) algorithm to allocate edge computing resources efficiently while ensuring trust and security.

## 3. System Model

**Figure 1:**
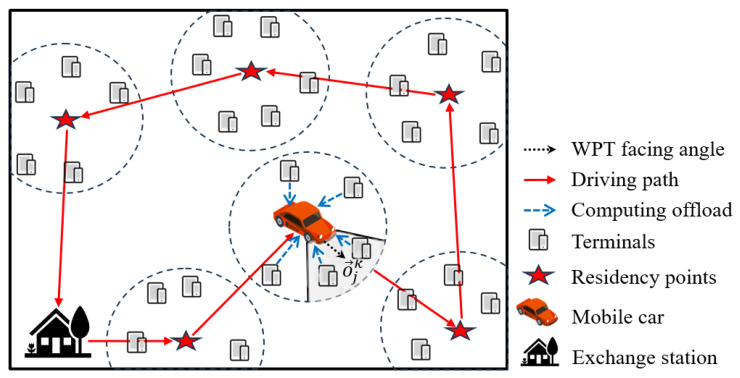
The overview of the system model.

The MEC system design for our oriented WPT is demonstrated in Figure 1. The scene model in this paper mainly consists of *M* wireless charging terminals, a mobile vehicle, and a battery swapping station. Therefore, mobile vehicles can wirelessly charge terminals within the coverage range of RF signals at a certain angle through RF transmitters. At the same time, mobile vehicles can also collect and calculate offloading tasks generated by terminals.

In the scenario, terminals can form *K* small cellular networks based on distance. These cellular network areas are all circles with a radius of *r*, which are also effective coverage areas for the radio frequency signals of mobile vehicles. In this paper, the center of the circle is used as the residence point for the mobile vehicle. Considering the impact of wireless charging transmission distance on system efficiency, we minimize this impact by optimizing the path planning of the mobile trolley and adjusting the transmit power and antenna configuration to ensure that the system can maintain efficient energy transfer and computing services under various operating conditions. Each time the mobile vehicle departs from the power exchange station, it travels through the planned shortest path to various residency points to provide directional wireless charging and computing offloading services for terminals, and finally, heads back to the power exchange station to replenish energy or prepare for the next task to be executed. The mobile vehicle provides battery replacement services at the battery swapping station in the scene. Owing to the mobility of the vehicle, our model is capable of accommodating diverse environmental conditions and requirements. Particularly in natural environments that require long-term or short-term monitoring, the mobile vehicle can provide the energy supply for the monitoring equipment and collect key data.

**Figure 2:**
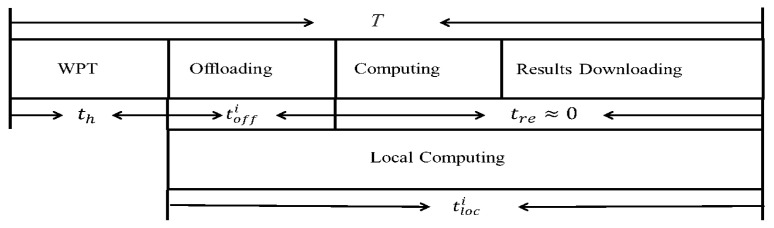
The first time slot allocation within the time block *T*.

**Figure 3:**
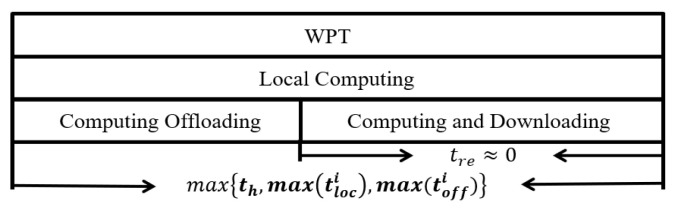
The second time slots allocation within the time block *T*.

The mobile vehicle can both receive offloading task data and transmit calculation results simultaneously. This paper considers two time-slot allocation schemes by reducing mutual interference between energy and data transmission channels, as well as integrating wireless charging modules, computing offloading, and local computing circuit modules separately into terminals. Figure 2 shows that a single time block *T* is demarcated into two phases, namely the WPT phase and the task data processing phase. Furthermore, Figure 3 shows that wireless charging, local computing, and data offloading can occur simultaneously when the power consumption per unit time is less than the collected energy. The mobile vehicle is equipped with a high-performance multi-core processor, and the calculation results are far less extensive than the scale of the calculation task. Therefore, the server calculation delay and calculation result return delay on the mobile vehicle can be ignored.

For a better understanding, this paper assumes that the mobile vehicle has prior knowledge and state information of CSI between itself and various terminal devices.

### 3.1. Energy Harvesting Phase

The power of directional WPT [33] can be formulated as,

(1) $$P_{0}=\left\{\begin{array}{cc} & \mu \frac{\cos\theta +c}{d+\gamma },0\leq d\leq r,-\frac{\pi }{2}\leq \theta \leq \frac{\pi }{2},\\ & 0,\mathrm{others}, \end{array}\right.$$

(2) $$d=||km_{i}\left|\right|,\theta =\arccos\frac{\vec{o_{j}^{k}}*\vec{m_{i}}}{|\vec{o_{j}^{k}}*\vec{m_{i}}|},r=\frac{\mu (\cos(\theta )+c)}{P_{\min}\eta t_{h}}-\gamma$$

where θ is the angle between the *j*-th orientation $\vec{o_{j}^{k}}$ of the mobile vehicle at the dwell point *k* and the angle $\vec{m_{i}}$ of the terminal device *i* relative to the dwell point *k*. *d* is the distance between the residence point *k* and the terminal device *i*. If this distance increases, it will cause the power received by the user to decrease. *r* represents the energy transmission range of the vehicle, which can be obtained from the minimum received power required by the terminal device $P_{min}$. λ is the wavelength of the signal. μ, *c*, γ are determined by the hardware parameters of the experimental environment and wireless charging device.

Hence, the energy harvested by terminal *i* within time $t_{h}$ can be formulated as

(3) $$E_{har}^{i}=\eta P_{0}ht_{h},\forall i\in M$$

This paper adopts the widely used block fading channel model in WPT-MEC systems, namely $h=10^{-3}d^{-\alpha }\varphi$. Also, φ indicates a short-term decline. The path loss index of the communication link is α. Given that the typical attenuation of signal power for all channels at 1 m is 30 dB, η is the energy conversion efficiency and η∈[0,1].

Given the finite battery capacity $C_{max,i}$ of terminal *i*, $E_{har}^{i}$ needs to meet:

(4) $$E_{har}^{i}\leq C_{max,i}-E_{res}^{i}-w\left(E_{off}^{i}+E_{loc}^{i}\right),\forall i\in M$$

where $E_{res}^{i}$ represents the remaining energy in the terminal *i* battery before charging; $E_{off}^{i}$ and $E_{loc}^{i}$ represent the energy consumed by terminal *i* for data offloading and local computing, respectively; when the value of *w* is 1, it indicates the time slot allocation that scheme 1 used, and when the value is 0, it indicates the time slot allocation that scheme 2 used.

### 3.2. Task Data Processing Phase

In this stage, the terminal performs task processing, including two parts: task data offloading and local computing.

#### 3.2.1. Offloading Model

This paper refers to a task model where task data can be arbitrarily segmented. According to Shannon’s formula, the offloading rate of the terminal *i* task is

(5) $$R_{i}=B_{i}\log_{2}\left(1+\frac{h_{i}P_{i}}{\sigma_{0}^{2}}\right),\forall i\in M,$$

where $B_{i}$ represents the bandwidth occupied by terminal *i*, as we adopt the FDMA scheme, which affects the total bandwidth $B_{max}$, which has constraints: $\sum_{i=1}^{ch}B_{i}\leq B_{max},\forall i\in \mathrm{ch}$, $h_{i}$ represents the channel gain that terminal *i* chooses to offload to the mobile vehicle; $P_{i}$ indicates the transmission power that terminal *i* chooses to offload; $\sigma_{0}^{2}$ denotes the additive Gaussian white noise’s power near the receiving end; ch represents the number of terminals within the effective WPT coverage range for a single orientation.

Assuming the total task volume of terminal *i* is $N_{i}\geq 0$ bits, the task of $N_{off}^{i}$ bits needs to be offloaded, so there are $0\leq N_{off}^{i}\leq N_{i},\forall i\in M,$

(6) $$N_{off}^{i}=R_{i}*t_{off}^{i},\forall i\in M$$

The energy required for offloading task data by terminal *i* is

(7) $$E_{off}^{i}=P_{i}*t_{off}^{i}+P_{c}*t_{off}^{i},\forall i\in M$$

wherein $P_{c}$ is the constant circuit power consumption of the terminal.

This paper uses $q_{i}$ to represent the CPU revolutions required for terminal *i* to calculate 1 bit of data. To ensure that the delay in result return can be ignored, assuming there are limitations: $\sum_{i=1}^{ch}N_{off}^{i}*q_{i}\leq Q,\forall i\in \mathrm{ch},$ where *Q* represents the computing power of the CPU of the edge server.

#### 3.2.2. Local Computation Model

After terminal *i* offloaded $N_{off}^{i}$ bits, local calculations are performed on the remaining bits:

(8) $$N_{loc}^{i}=N_{i}-N_{off}^{i},\forall i\in M.$$

Thus, the time allocated for local calculation by terminal *i* can be computed:

(9) $$t_{loc}^{i}=\frac{N_{loc}^{i}*q_{i}}{f_{i}},\forall i\in M,$$

where $f_{i}$ represents the CPU frequency of terminal *i*, which cannot exceed the maximum frequency limitations on $f_{max}^{i}$.

At each time block, terminal *i* starts allocating task data after collecting energy or at the same time as collecting energy. Based on the above-mentioned computation offloading and local computation processes, time constraints can be obtained: $w*\left(t_{h}+max\left(t_{off}^{i},t_{loc}^{i}\right)\right)+\left(1-w\right)*max\left(t_{h},t_{off}^{i},t_{loc}^{i}\right)\leq T,\forall i\in M$.

The energy required for local processing is calculated through:

(10) $$E_{loc}^{i}=N_{loc}^{i}*q_{i}*e_{i},\forall i\in M,$$

where $e_{i}=k_{i}f_{i}^{2}$ represents the energy consumption generated by the CPU of terminal *i*, and $k_{i}$ represents the effective capacitance coefficient of terminal *i*.

Based on the above computing offloading and local calculation processes, there are constraints $E_{off}^{i}+E_{loc}^{i}\leq E_{res}^{i}+E_{har}^{i},\forall i\in M.$

## 4. Problem Formulation

We propose a relaying cooperative offloading scheme among terminal devices to address the dual near-far effect. Table 1 illustrates the variables, accompanied by their descriptions.

This paper’s model sets out to minimize the weighted aggregate of system latency and energy consumption for every terminal tasks, as represented by the formula:

(11) $$\min\beta *\sum_{k=1}^{M}\sum_{j=1}^{A}\max\left(t_{h},\max\left(t_{off}\right),\max\left(t_{loc}\right)\right)+\left(1-\beta \right)*\sum_{i=1}^{M}\left(E_{off}^{i}+E_{loc}^{i}\right)$$

s.t.

(12) $$\begin{array}{cc} \; & C1:E_{off}^{i}+E_{loc}^{i}\leq E_{res}^{i}+E_{har}^{i},\forall i\in M\\ \; & C2:E_{off}^{i}=P_{i}*t_{off}^{i}+P_{c}*t_{off}^{i},\forall i\in M\\ \; & C3:\sum_{i=1}^{ch}B_{i}\leq B_{max},\forall i\in \mathrm{ch}\\ \; & C4:0\leq N_{off}^{i}\leq N_{i},\forall i\in M\\ \; & C5:w*\left(t_{h}+max\left(t_{off}^{i},t_{loc}^{i}\right)\right)+\left(1-w\right)*max\left(t_{h},t_{off}^{i},t_{loc}^{i}\right)\leq T,\forall i\in M\\ \; & C6:\sum_{i=1}^{ch}N_{off}^{i}*q_{i}\leq Q,\forall i\in \mathrm{ch}\\ \; & C7:f_{i}\leq f_{max}^{i},\forall i\in M \end{array}$$

In (11), β represents the weight of the system delay of all terminals in the scene whose value is 1 when combined with the weight of system energy consumption; $t_{off}$ and $t_{loc}$ record the offloading time and local computation time of all terminals within the effective WPT coverage range for a single orientation; *A* represents the total count of orientations of the mobile vehicle at each parking point.

Since we have divided the problem into several stages, such as device clustering, path planning, and optimization objective solving, the decisions made in each stage will affect the subsequent ones. Moreover, the problem involves many variables, including wireless power supply time, computation offloading, local computing task scheduling, and system bandwidth scheduling. There are complex interdependencies among these variables, and their interactions must be considered. The problem is classified as NP-hard due to the above characteristics, particularly the multivariate combinatorial optimization and the tight coupling between variables [34,35]. In tackling this challenge, the following algorithm has been designed.

## 5. The IDDPG-Based Resource Scheduling Algorithm

This section elaborates on the solution for the system model proposed above, which is divided into three parts: device clustering, path planning, and solution algorithms during task execution.

### 5.1. Device Clustering

Before performing task offloading, we should cluster all terminal devices in an area according to their physical locations, which are randomly distributed. It is divided into several circular networks with the radius of the effective coverage distance of the radio frequency signal of the mobile vehicle so that the mobile vehicle can effectively interact with the nearby terminal equipment. Construing this process as clustering, it involves organizing circular regions by their distances from data points, a challenge that the K-means algorithm is particularly adept at [36].

The K-means algorithm aims to categorize data set samples into K-distinct clusters. It identifies each cluster’s centroid as the mean of its constituent data points. The objective is twofold: to minimize the distances of data points to their respective centroids and to maximize the distance between centroids of separate clusters [37]. The detailed procedure is outlined below:

- As the K-means algorithm begins, *K* points from the data are randomly appointed as provisional centers. These initial centers serve as the starting points for the algorithm, initiating the iterative process that will shape the clusters.
- In the assignment phase, the K-means algorithm proceeds by evaluating each point in the dataset, calculating its distance to every existing centroid. The Euclidean distance is commonly employed for this measurement. Based on these computations, data points are distributed among clusters by identifying the nearest centroid for each, thus refining the cluster memberships.
- Then, once all data points have been assigned to their respective clusters, the algorithm continues to recalculate the centroids by averaging the data points within each cluster. This phase is crucial to the K-means algorithm, as it reshapes the clusters’ geometrical form and redistributes their positions in the feature space.
- In its iterative process, the K-means algorithm assigns data points to clusters and updates the centroids accordingly. During successive iteration, the allocation of data points to clusters is reconsidered in light of the current centroids, and the centroids are repositioned to reflect the mean location of their respective data points. This iterative process continues until the assignments of data points to clusters stabilize, indicating that the algorithm has converged. Upon completion, the algorithm provides *K* clusters, each characterized by its centroid and the set of data points it encompasses.
  For each sample point *i* in the dataset, the contour coefficient is calculated as follows:

(13) $$s\left(i\right)=\frac{b(i)-a(i)}{\max\{a(i),b(i)\}}$$

(14) $$\bar{s}=\frac{1}{n}\sum_{i=1}^{n}s\left(i\right)$$

where a(i) denotes the average distance between point *i* and other points within the same cluster, indicating the cohesion degree within the cluster. Conversely, b(i) represents the average distance between point *i* and all points in the nearest cluster, signifying the separation degree between clusters. For the entire dataset, the average silhouette coefficient, denoted as $\bar{s}$, can be calculated by computing s(i) for all sample points *i* and then taking the average, where *n* is the total number of sample points in the dataset.

When using the k-means algorithm, choosing an appropriate *K* value is very critical [38]. We have integrated a binary search method into the K-means algorithm, resulting in the BK-means algorithm. This enhanced algorithm searches for the effective *K* value on the premise that the communication distance of the mobile vehicle is satisfied, and the size of the circle region after clustering is just less than or equal to the area defined by the radius of the mobile vehicle’s communication reach. Then, the clustering results are evaluated by the contour coefficient, a measure used in cluster analysis to assess the quality of the clustering, particularly in spatial or geometric contexts. This coefficient determines how well the clusters are formed by an algorithm corresponding to the underlying structure of the data. The larger the contour coefficient is, the more reasonable the clustering of logarithmic points is [39]. Finally, the clustering results with the maximum contour coefficient are obtained by running the clustering algorithm several times.

### 5.2. Path Planning

The clustering centers are determined by clustering the terminal devices in the dynamic scene. These centers are the ideal residence points of the vehicle. To plan the vehicle’s course efficiently and reasonably, the physical position of the charging station is set at the origin coordinate. The vehicle starts from the charging station and traverses all the stopping points according to the established strategy. Finally, the vehicle returns to the charging station for electrical energy replenishment. This process constitutes a typical path-planning problem aimed at maximizing resource utilization.

We approach the path-planning problem as a variant of the generalized Traveling Salesman Problem (TSP). We utilize the location coordinates of cluster centers and charging stations as the city coordinates within the TSP framework and transform the problem into finding the shortest closed route such that each city is visited just once by the traveling salesman, with the trip concluding at the starting location. Our objective is to minimize the driving path length and aim to realize the most efficient energy consumption and the shortest travel time for the vehicle [40].

**Figure 4:**
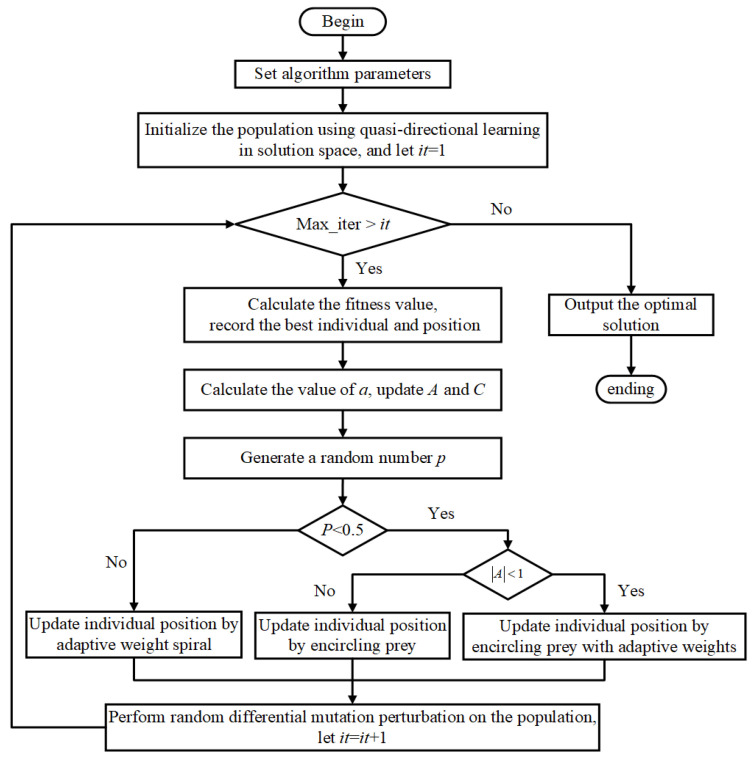
The flowchart of the hybrid whale–bat optimization algorithm.

To effectively plan the optimal path among these stations, a hybrid Whale Optimization Algorithm is employed. A metaheuristic technique that mirrors the predatory actions of humpback whales and has garnered attention due to its excellent search capability and rapid convergence. We integrate an adaptive strategy into the algorithm, which makes real-time adjustments to parameters using feedback from the search process, thereby making the path search more flexible and efficient. Adaptively adjusting the search range and step size allows WOA to balance exploration and exploitation [41], avoid local optima, and ultimately discover the global optimal or near-optimal path. Figure 4 presents the detailed flow of the algorithm we have introduced.

### 5.3. IDDPG Algorithm

After device clustering and path planning, the next step is to address the proposed optimization objectives. In this scenario, the optimization of total system latency and energy usage for all terminal devices performing tasks involves variables such as wireless energy supply time, computing offloading, local computing task scheduling, and system bandwidth allocation. The choice between parallel and serial interactions complicates the research on interactions between mobile vehicles and terminal devices. Furthermore, the variables involved are tightly coupled, rendering the problem a mixed-integer nonlinear programming (MINP) issue. More importantly, these variables must adapt swiftly and appropriately to shifts in the system’s environmental parameters. It is evident that the problem studied is a multi-variable combinatorial optimization issue, and these variables are constrained within specific value ranges. Consequently, this problem is also classified as an NP-hard problem. To tackle the issue discussed within this research, DDPG combined with an immune differential process is designed, and this algorithm is recorded as the IDDPG algorithm.

#### 5.3.1. State Space

Within the WPT-MEC environment, the state space is influenced by the vehicle, *M* terminal devices, and the environment. The *i*-th time slot’s system state is detailed as:

(15) $$s_{i}=\left(E_{battery}\left(i\right),q\left(i\right),p_{1},...,p_{k}\left(i\right),D_{remain}\left(i\right)\right)$$

where $E_{battery}\left(i\right)$ is the residual power of the vehicle in the *i*-th slot, q(i) is the position of the mobile vehicle, $p_{M}\left(i\right)$ is the position information of the terminal device *M*, $D_{remain}\left(i\right)$ denotes the task information that the terminal devices require processing by the mobile vehicle.

#### 5.3.2. Action Space

The action space determines all of the potential actions that the mobile vehicle can make in each state. We need to consider the continuity and diversity of actions, while ensuring that actions are selected to maximize the overall performance. The action $a_{i}$ can be denoted as:

(16) $$a_{i}=\left(U\left(i\right),R_{M}\left(i\right)\right)$$

where U(i) denotes the orientation angle of the vehicle, and $R_{M}\left(i\right)$ is the task-offloading proportion for the *M* terminal devices. $U\left(i\right)\in \left[0,2\pi \right],R_{M}\left(i\right)\in \left[0,1\right]$. By adjusting the orientation, the vehicle can optimize the service coverage and quality, thus maximizing the energy transfer efficiency and ensuring more efficient energy delivery to the end devices.

#### 5.3.3. Reward Function

The reward function is a reflection of the instant gain the agent experiences from a particular action, coupled with the sum of rewards over time, leading the agent to explore the environment and optimize its behavior policy. Therefore, choosing an appropriate reward function is crucial for the agent to learn the best policy.

In this model, it is particularly critical to design an appropriate reward function that guides the agent in learning to achieve our goal while meeting the task requirements of the terminal devices. Our reward function is formulated as follows:

(17) $$r_{i}=r\left(s_{i},a_{i}\right)=f\left(i-1\right)-f\left(i\right)$$

where f(i) denotes the objective function’s value at time *i*,

(18) $$f\left(i\right)=\beta *\sum_{k=1}^{M}\sum_{j=1}^{A}\max\left(t_{h},\max\left(t_{off}\right),\max\left(t_{loc}\right)\right)+\left(1-\beta \right)*\sum_{i=1}^{M}\left(E_{off}^{i}+E_{loc}^{i}\right)$$

We construct the reward function as the variance in objective function values at the previous and present times. If this difference is positive, it indicates that the weighted aggregate of the terminal devices’ total delay and energy consumption has decreased from the last interval to the present moment. Having identified a more efficient strategy, a suboptimal result from the agent’s policy is penalized with a negative reward. This negative feedback serves to discourage the agent from repeating such strategies, thereby guiding it toward more efficient decision-making.

The DDPG algorithm acquires actions by incorporating a differential immune process into the neural network model during the training and learning stages, which are obtained through the combined action of the magic function, namely the actor network and the differential immune process [42]. Then, the intelligent agent is optimized to obtain a better decision plan. Algorithm 1 shows the pseudocode of the IDDPG algorithm.
**Algorithm 1:** The procedure of IDDPG$\theta^{Q}$ and $\theta^{\mu }$ randomly initialize the Critic network $Q(s,a|\theta^{Q})$ and Actor network $\mu (s|\theta^{\mu })$
$\theta^{Q^{\prime }}\leftarrow \theta^{Q},\theta^{\mu^{\prime }}\leftarrow \theta^{\mu }$. Initialize target network weight parameters $Q^{\prime }$ and $\mu^{\prime }$
Initialize experience replay area *R*
**for** episode=1,...,M **do**:      Action exploration, random noise *N* initialization      Obtain initial observation state $s_{1}$      **for** t=1,...,T **do**:            Through the Actor network generate actions $a1_{t}=\mu \left(s_{t}|\theta^{\mu }\right)+N_{t}$            Immune differential process generates actions $a2_{t}$            Execute action $a1_{t}$ and $a2_{t}$            Received reward $r1_{t}$ and $r2_{t}$ and environmental status $s1_{t+1}$ and $s2_{t+1}$            Take a set of data with a larger reward as the final action $a_{t}$            Final reward $r_{t}$ and environmental status $s_{t+1}$            Data $\left(s_{t},a_{t},r_{t},s_{t+1}\right)$ stored in R            Randomly sample a multidimensional array $\left(s_{i},a_{i},r_{i},s_{i+1}\right)$ of batch numbers *N* from *R*                                      $y_{i}=r_{i}+\gamma Q^{\prime }\left(s_{i+1},\mu^{\prime }\left(s_{i+1}|\theta^{\mu^{\prime }}\right)|\theta^{Q^{\prime }}\right)$            Minimize the loss function *L* to update the Critic network:                                      $Loss=\frac{1}{N}\sum_{i}{\left(y_{i}-Q\left(s_{i},a_{i}|\theta^{Q}\right)\right)}^{2}$            Sampling strategy gradient update Actor strategy network:                                      $\nabla_{\theta^{\mu }}J\left(\theta^{\mu }\right)≅\frac{1}{N}\sum_{i}\nabla_{\theta^{\mu }}\mu \left(s_{i}|\theta^{\mu }\right)\nabla_{a}Q\left(s_{i},a|\theta^{Q}\right){|}_{a=\mu (s_{i})}$            Update target network:                                      $\theta^{Q^{\prime }}\leftarrow \tau \theta^{Q}+\left(1-\tau \right)\theta^{Q^{\prime }};$                                      $\theta^{\mu^{\prime }}\leftarrow \tau \theta^{\mu }+\left(1-\tau \right)\theta^{\mu^{\prime }}$      **end for****end for**

## 6. Numerical Simulation

The mobile vehicle employs electromagnetic-resonance wireless energy transfer technology to power the terminal device wirelessly through an RF transmitter [43]. In this system, we utilized a 500 W RF transmitter (e.g., Spark Connected Yeti) with an 0.8 energy conversion efficiency for electromagnetic resonance-based wireless power transfer [44]. Multiple resonance coils serve as transmitters, while receivers are resonant coils that harvest energy from the electromagnetic field. To optimize transfer efficiency, a matching network aligns the transmitter and receiver impedances [45]. The system includes an energy storage unit for storing captured energy and can scale from watts to kilowatts (Table 2).

Simulation experiments are formulated to test the proposed algorithm’s validity within our model. Channel parameters include a path loss index of 2, where the position of the terminal device *i* is situated randomly within a 100 m square area, and the data task size for terminal device *i* follows a uniform distribution pattern. This paper compares and simulates the arithmetic optimization algorithm (AOA), mayfly algorithm (MA), and DQN, proving the superiority of this algorithm. All emulators are written in the Python programming language.

**Figure 5:**
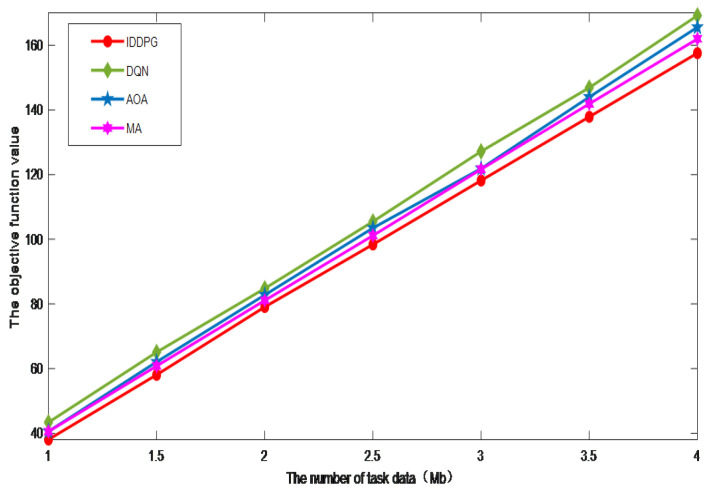
The impact of task data volume.

Figure 5 illustrates the correlation between the number of different terminal tasks and the corresponding objective function values for the four studied algorithms. We can intuitively observe that the objective function value exhibits a growing trend with the increase in the number of terminal tasks. This phenomenon is present across all four algorithms, but the rate of increase and the peak values reached vary. Among these algorithms, the IDDPG algorithm demonstrates the best performance. It maintains a lower growth rate in the objective function value and can sustain good performance even as the number of terminal tasks increases. This indicates that the IDDPG algorithm can effectively balance the requirements of different tasks and find more optimal solutions when dealing with complex optimization problems. In contrast, the DQN algorithm in deep reinforcement learning performs relatively poorly compared to the others. The DQN algorithm typically excels in problems with discrete action spaces but struggles with continuous control problems. This limitation arises because, when the DQN algorithm makes decisions, the value of each action selected by the agent can only be discrete, which restricts its flexibility and precision in continuous action spaces. In continuous control problems, the quality of the optimization results largely depends on the action values, and the DQN algorithm may not be precise enough in evaluating these continuous action values, leading to a degradation in overall performance.

**Figure 6:**
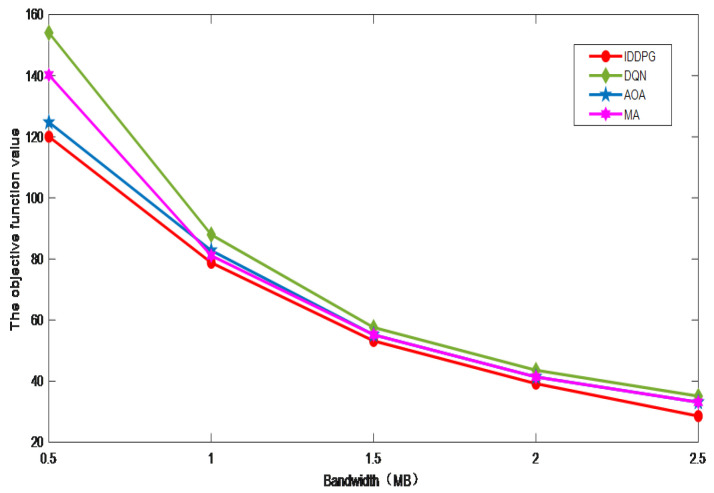
The impact of system bandwidth.

Figure 6 presents a comparative analysis of experimental outcomes across varying bandwidth conditions and illustrates the impact of bandwidth allocation on system performance metrics. It shows that the objective function decreases as the bandwidth increases. This is because, given the same data task and energy consumption for offloading, occupying a larger bandwidth allows the terminal device to offload at a faster rate, thereby reducing the offloading time for data tasks. As a result, it is possible to decrease the overall processing delay of the system. The IDDPG algorithm has once again demonstrated its superior performance. By employing an intelligent resource scheduling strategy, it can effectively utilize the available bandwidth and identify an optimal resource allocation plan. This plan not only minimizes the final objective function value but also enhances the efficiency of resource usage, thereby further optimizing system performance. These attributes highlight the IDDPG algorithm’s significant adaptability and robustness in dynamic environments, particularly when resources are constrained.

**Figure 7:**
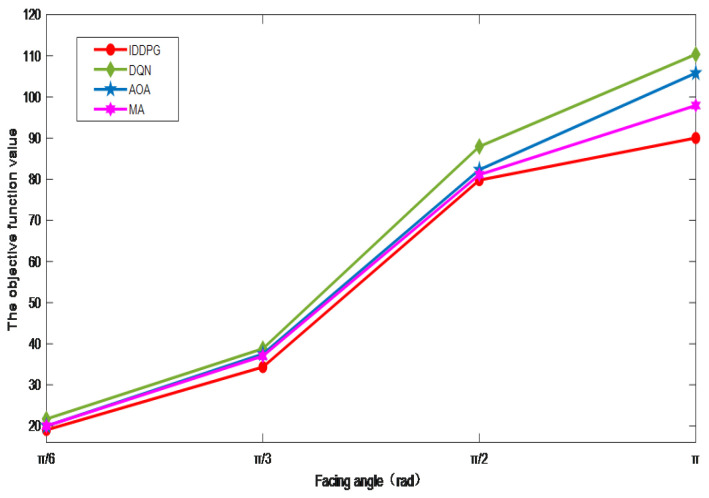
The impact of facing angle.

The experimental outcomes as influenced by the changes in effective charging angles are presented in Figure 7. It shows that as the effective charging angle of the mobile vehicle increases from π/6 to π, the objective function value shows an increasing trend. This is because although the increase in the effective charging angle of the mobile vehicle will enable more terminals to charge wirelessly at the same time, when the distance between the terminal and the mobile vehicle is equal, the position of the terminal deviates more from the direction of the mobile vehicle’s front ray. The lower the efficiency of wireless charging, the more time it takes for wireless charging to increase significantly.

**Figure 8:**
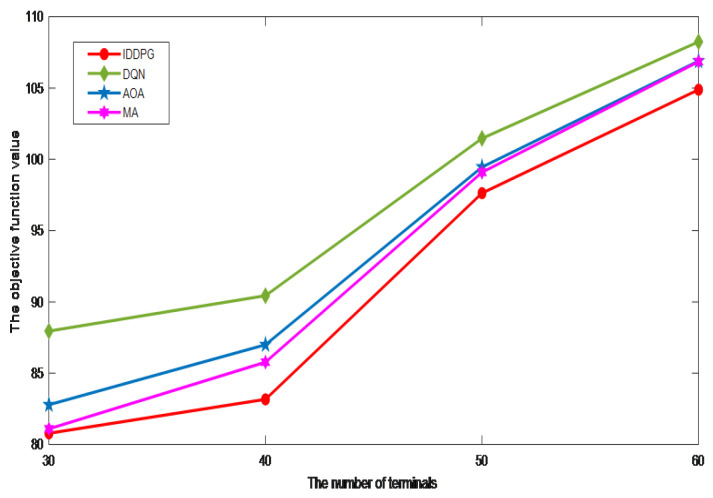
The impact of the number of terminals.

Figure 8 considers the scenario with varying numbers of terminal devices and shows that the objective function value exhibits an increasing trend as the number of terminals increases. The visual data indicate that as the terminal count expands, the optimization results of the DQN algorithm, AOA algorithm, and MA algorithm are constantly approaching, and the gap gradually decreases. However, the optimization effect of the DQN algorithm still cannot surpass other algorithms, while the AOA algorithm gradually catches up with the optimization effect of the MA algorithm. The outstanding performance of IDDPG in multi-device scenarios is due to its ability to flexibly handle both continuous and discrete action spaces and to maintain a good balance between exploring new strategies and exploiting existing knowledge. This makes it surpass other algorithms in optimizing the objective function and improving system performance.

**Figure 9:**
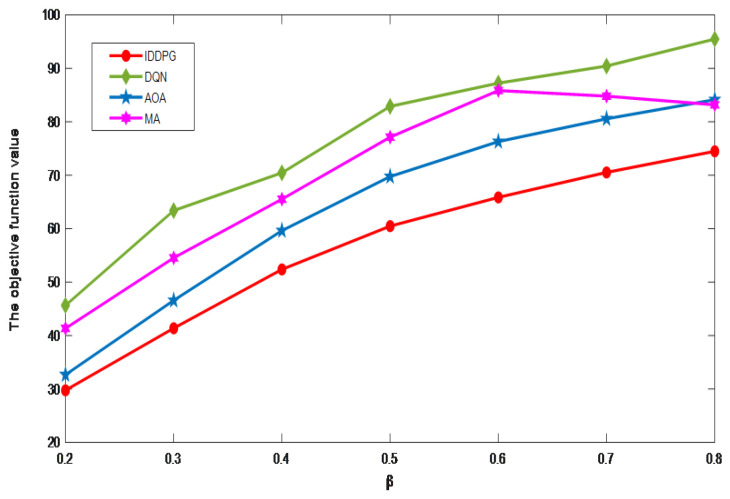
The impact of the β value.

Figure 9 investigates the effects of varying β values on the objective function. The gradual increase in delay is paralleled with the objective function’s value during the rise. While our method puts energy consumption and delay at the same order of magnitude, the calculation result of delay is larger than that of energy consumption. The graph illustrates that the interval between the optimization results of the four algorithms is significantly larger. The optimization performance of the IDDPG algorithm still leads the other three, and with the β value increase, the gap between the optimization results of the AOA algorithm becomes larger and larger (Table 3).

## 7. Conclusions

This paper presents a directional WPT-MEC system model featuring multiple terminals, which collect energy from the RF signals transmitted by a mobile vehicle, store it in batteries, and use it for processing tasks. In this model, FDMA technology is used to achieve task offloading for multiple terminals simultaneously. We aim to enhance the allocation of energy, communication, and computing resources through joint optimization, minimizing the weighted aggregate of task execution delay and energy consumption of terminals. To optimize the efficiency of solving, we applied the DDPG algorithm to this paper and made improvements, proposing the IDDPG algorithm. The simulation outcomes indicate that the IDDPG algorithm proposed herein excels over three alternative algorithms. It is adept at finding optimal resource allocation strategies and offers enhanced stability in convergence. In the future, we will validate the practicality of our solution and the effectiveness of the IDDPG algorithm in actual scenarios using existing directional energy emitters and wireless charging devices.

## Figures and Tables

**Table 1:** List of notations.

Symbol	Description
*K*	number of cellular networks
*T*	the length of the time block
$B_{\max}$	system bandwidth
ω	number of time slot schemes selected
*A*	the count of terminals in the valid WPT range for each trolley
θ	the angle between the *j*-th orientation $\vec{o_{j}^{k}}$ of the mobile vehicle and the angle $\vec{m_{i}}$ of the
	terminal device *i* relative to the dwell point *k*
*d*	the distance between the residence point *k* and the terminal device *i*
$E_{har}^{i}$	the energy collected by terminal *i* within time $t_{h}$
$E_{res}^{i}$	the remaining energy in the terminal *i* battery before charging
$E_{off}^{i}$	terminal *i*’s energy consumption during data offloading
$E_{loc}^{i}$	terminal *i*’s energy consumption during local computing
$B_{i}$	the bandwidth occupied by terminal *i*
$h_{i}$	the selected channel gain for offloading from terminal *i* to the mobile vehicle
$P_{i}$	terminal *i*’s selected transmission power for data offloading
$\sigma_{0}^{2}$	the additive Gaussian white noise’ power near the receiving end
$\sigma^{2}$	the noise power near either receiving end
ch	the number of terminals within the effective WPT coverage range for a single orientation
$P_{c}$	the terminal’s constant circuit power consumption
$f_{i}$	the processing frequency of terminal *i*’s CPU
$f_{\max}^{i}$	the upper limit of CPU speed
$k_{i}$	the effective capacitance coefficient of terminal *i*
$e_{i}$	the energy consumption generated by the CPU of terminal *i*
$q_{i}$	the CPU revolutions required for terminal *i* to calculate 1 bit of data
*Q*	the computing power that the CPU of the edge server
$Q_{1}$	the maximum computing power provided by the edge server

**Table 2:** Simulation parameters.

T=5 s	$B_{\max}$ = 50 MHz	$\sigma^{2}=10^{-9}$ W
μ=3.893	c=0.1161	γ=0.1
$Q_{1}=2\times 10^{10}$ cycles	$f_{\max}^{i}$ = 1.5 GHz	$P_{c}=10^{-6}$ W
$C_{\max}^{i}$ = 6 J	$k_{i}\in \left[10^{-29},10^{-27}\right]$	$N_{i}\in \left[0,4\right]$ Mb

**Table 3:** List of notations.

Symbol	Description
MEC	mobile edge computing
WPT	wireless power transfer
AWOA	whale optimization algorithm with adaptive mechanism
IDDPG	immune differential enhanced deep deterministic policy gradient
IoT	Internet of Things
FDMA	frequency division multiple access
WOA	whale optimization algorithm
DDPG	deep deterministic policy gradient
RL	reinforcement learning
DRL	deep reinforcement learning
SWIP	simultaneous wireless information and power transfer
NOMA	integrated non-orthogonal multiple access
MIMO	multiple-input multiple-output
ADMM	alternating direction method of distributed multipliers
IRS	intelligent reflecting surface
SCNs	small cell networks
ACCRA	admission control and computation resource allocation
UAV	unmanned aerial vehicle
DQN	deep Q-Network
MINLP	mixed integer nonlinear programming
CSI	channel state information
A3C	asynchronous advantage actor-critic
TSP	traveling salesman problem
AOA	arithmetic optimization algorithm
MA	mayfly algorithm

## Data Availability

Data is contained within the article.
